# A Technology Aid to Help People with Blindness and Moderate Intellectual Disability Retrieve Common Objects from Storage Units: A Proof-of-Concept Study

**DOI:** 10.3390/s24144453

**Published:** 2024-07-10

**Authors:** Chiara Filippini, Giulio E. Lancioni, Gloria Alberti, Francesco Pezzuoli, Patrizia Ceccarani

**Affiliations:** 1 Lega F. D’Oro Research Center, 60027 Osimo, Italy; giulio.lancioni@uniba.it (G.E.L.); alberti.g@legadelfilodoro.it (G.A.); ceccarani.p@legadelfilodoro.it (P.C.); 2 Department of Design, University of Camerino and LiMiX Srl, 63100 Ascoli Piceno, Italy; francesco.pezzuoli@unicam.it

**Keywords:** technology, tags, tag reader, smartphone, blindness, intellectual disability, object retrieval

## Abstract

Background: People with blindness and intellectual disability can have problems locating, identifying, and retrieving objects needed for daily activities (e.g., clothes and food items) from familiar storage contexts, such as cupboards and cabinets. Objective: This preliminary study assessed a technological system designed to help three people with those problems improve their performance. Methods: The technological system, which involved the use of tags with radio frequency identification codes, a smartphone, and a tag reader, aimed to guide the participants in searching and retrieving objects from three different storage units. In practice, the system provided different feedbacks depending on whether the participants were searching (a) in a wrong storage unit, (b) in a wrong shelf/drawer of the right storage unit, or (c) in the right shelf/drawer of the right storage unit. Results: All participants were successful in retrieving objects correctly with the technological system. The results also showed that (a) the participants preferred using the system over a control strategy, (b) were able to switch on and off the system independently, and (b) staff rated the system positively. Conclusions: These preliminary findings suggest that the system might be a useful support tool for people with blindness and intellectual disability.

## 1. Introduction

People with blindness and intellectual disability tend to face problems in main areas of their daily life, such as orientation and traveling, and performance of occupational and vocational tasks [1,2,3,4,5,6]. Major intervention efforts have been made over the years to support them in those areas [2,7,8,9,10,11]. The same people may also have problems in dealing with more basic/specific forms of engagement, such as locating, identifying, and retrieving objects needed for daily activities (e.g., clothes and food items) from familiar storage contexts, such as cupboards and shelving units [12,13,14,15]. These latter problems may be due to two main factors that can also act in combination. First, people may not remember the specific location of a number of objects and thus may find it difficult to search for and eventually find them [12,14,16,17]. Second, some objects are very similar to others (e.g., food items, such as single-dose sachets of oil and vinegar, and clothes, such as short- and long-sleeve shirts) and thus are difficult or even impossible to discriminate on tactile inspection [18,19,20,21].

No systematic research efforts have apparently been made to identify effective ways of (to devise effective intervention means for) alleviating problems in locating, identifying, and retrieving objects. In these situations, two basic strategies seem to be commonly applied to support people’s performance. One such strategy involves the use of mini object replicas as signaling cues [4,21]. For example, mini object replicas could be fixed outside and/or on the shelves of cupboards and cabinets as cues to signal which objects can be found inside or on each shelf of those storage places. A similar strategy involves the use of stickers with words written in Braille instead of mini object replicas [22].

While no data are available on the overall effectiveness of those strategies, the general assumption is that they may be helpful and facilitate people’s task of locating and retrieving objects. Some recent research has hinted at the possibility of using technology solutions as an addition (or alternative) to the aforementioned strategies. Specifically, studies suggested that near-field communication (NFC) technology might be used to help people with blindness or low vision recognize familiar objects [23,24,25].

To make progress in this area, one might advocate the development of a specific technology system aimed at helping people to reduce the impact of the problem in an effective and friendly manner [13,26,27]. The technological system could be designed to (a) ensure that people identify and start searching in the correct storage context (e.g., cupboard), (b) guide people in the search within such a storage context, and (c) provide people with feedback as soon as they reach the objects that they were set to find.

This preliminary (proof-of-concept) study was aimed at assessing such a technological system with three adults who were diagnosed with blindness and moderate intellectual disability. The technological system entailed the use of tags with radio frequency identification codes suitable for NFC, a smartphone, and a tag reader, which was linked to a smartphone via Bluetooth using a dedicated application. The tag reader was a simplified version of the Talking Hands device recently reported [28]. This tag reader was selected since a thorough search for NFC readers on the market did not identify devices that could be worn comfortably by the participants of this study. The tag reader was set to read the aforementioned tags and send the relevant data to the smartphone, which was instrumental in providing the participants with feedback/guidance.

For each participant, the assessment of the system involved a comparison of its effects in helping to retrieve familiar objects with the effects of one of the traditional strategies mentioned above (i.e., use of mini object replicas or use of stickers with words in Braille).

## 2. Materials and Methods

### 2.1. Participants

The three participants, who are here reported with the pseudonyms of Travis, Asher, and Lucas, were 55, 51, and 64 years of age, represented a convenience sample [29] and shared the same difficulties in finding and retrieving objects (see below). They were totally blind and had a diagnosis of intellectual disability. Although no recent intelligence testing had been carried out and no IQ scores were available, all three participants were rated to function within the moderate intellectual disability range. The rating was provided by the psychological services of the rehabilitation and occupation centers that the participants attended. Their Vineland age equivalents assessed by using the second edition of the Vineland Adaptive Behavior Scales [30,31] were (a) 5 years and 3 months, 4 years and 5 months, and 4 years and 6 months, respectively, for Daily Living Skills (personal sub-domain), and (b) 6 years and 2 months, 5 years and 6 months, and 5 years and 1 month, respectively, for Receptive Communication.

They were recruited for the study based on four conditions. First, they had difficulties in finding/retrieving familiar objects relevant to their daily lives due to problems in locating and discriminating those objects within the related storage units (e.g., cupboards, cabinets) and/or identifying the storage units in which those objects were located. Second, they had the motor and language reception skills needed for using the technological system to be assessed in this study. Third, they had expressed their willingness to be involved in the study and use such a system, which had been presented to them in advance. Fourth, staff considered a support technology to help the participants in locating/retrieving objects highly desirable and were in favor of this study.

### 2.2. Ethical Approval and Informed Consent

The participants had expressed their willingness to be involved in this study, but were unable to read and sign a formal consent document authorizing them to do that. To ensure a respectful and transparent recruitment procedure, the participants’ legal representatives were asked to (a) confirm the participants’ willingness to be involved, and (b) take care of the consent process by reading and signing the consent forms on the participants’ behalf. This study complied with the 1964 Helsinki Declaration and its later amendments and was approved by an institutional Ethics Committee.

### 2.3. Setting, Storage Units, Objects, Searching and Retrieving Task, Sessions, and Research Assistants

This study was carried out in a large room of the rehabilitation and occupation facilities that the participants attended. The room was supplied with a desk and three storage units (i.e., a cupboard, a cabinet, and a drawer unit, similar to those used by the participants in their daily contexts). The units had six shelves or six drawers containing familiar objects. Objects of three different categories, including food and drinks, clothes, and personal hygiene and grooming (Travis and Lucas), or food and drinks, clothes, and occupational, and vocational activities (Asher), were used across different sessions. Within each session, 18 different types of objects were distributed in the three storage units (i.e., six types per unit). For every type of object (e.g., pasta) several exemplars/packs were available (as would be expected in a daily situation). For example, the top (sixth) shelf of the cabinet could contain five pasta packs. The fifth shelf could contain three biscuit packs. The fourth shelf could contain four boxes with single-dose sachets of oil. The third shelf could contain four boxes of single-dose sachets of vinegar. The second shelf could contain three bottles of natural water. The first shelf could contain three bottles of sparkling water. A similar arrangement was used for the six types of objects available in the cupboard and the six types of objects available in the drawer unit. The task the participants were to carry out within a session was to search and retrieve eight objects out of those available in that session. The objects to be searched and research assistants’ request sequences changed across sessions.

Typically, the participants received two sessions per day 3 to 6 days a week. The sessions lasted the time needed for the participants to retrieve the eight objects that the research assistants asked them to find and bring to the desk available in the room in front of the three storage units.

Three research assistants were involved in the implementation of the study sessions and data recording. They held a University degree, were experienced in using technology-aided intervention programs with persons with disabilities, and were familiar with data-recording procedures.

### 2.4. Technology System

The technology system involved tags with radio frequency identification codes suitable for NFC, a tag reader, and a smartphone equipped with two applications: the Talking Hands application dedicated to recognizing the tags’ codes and the MacroDroid application, which controlled the verbalization of appropriate messages.

**Figure 1:**
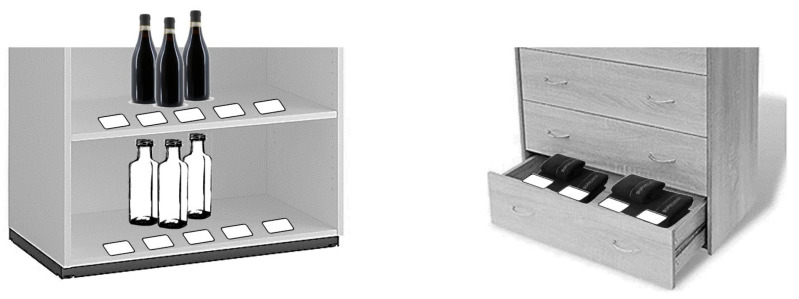
Schematic representation of sections of two of the storage units showing the position of the tag series.

**Figure 2:**
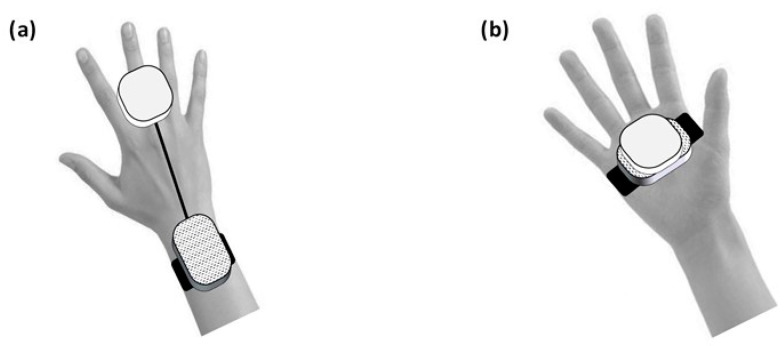
Schematic representation showing where the sections of the tag reader were fixed (**a**) during the intervention and (**b**) during the system wearing test.

The tags (5.4 × 8.5 × 0.1 cm; NCF MIFARE RFID, 1 K 13.56 MHz) were fixed on the single shelves of the cupboard and cabinet and the single drawers of the drawer unit. Specifically, each shelf and each drawer was fitted with a series of four or five tags, one next to the other (see Figure 1 for an illustration). The tags of each series had the same code number, which was recognizable by the tag reader. The tag reader consisted of two watch-like sections (of 6.5 × 4 × 1.5 cm and 4.5 × 4.5 × 0.9 cm). During the intervention phase of the study, one section was strapped on the participant’s wrist and the other on the back of the participant’s fingers (see Figure 2a). The section on the wrist included an electronic circuit, which was designed to send Bluetooth signals to the smartphone. The other section included an NFC module designed to recognize the identification code of the tag series. During the final (system wearing) test, the two sections were glued together and fixed to an elastic band so that the participants could put the system on and off independently (see Figure 2b).

The MacroDroid is a practical automation application for the creation of macros within Android devices. It was programmed to (a) combine tag codes and object names and (b) provide the participants with specific feedback messages during their searching performance (see below).

**Figure 3:**
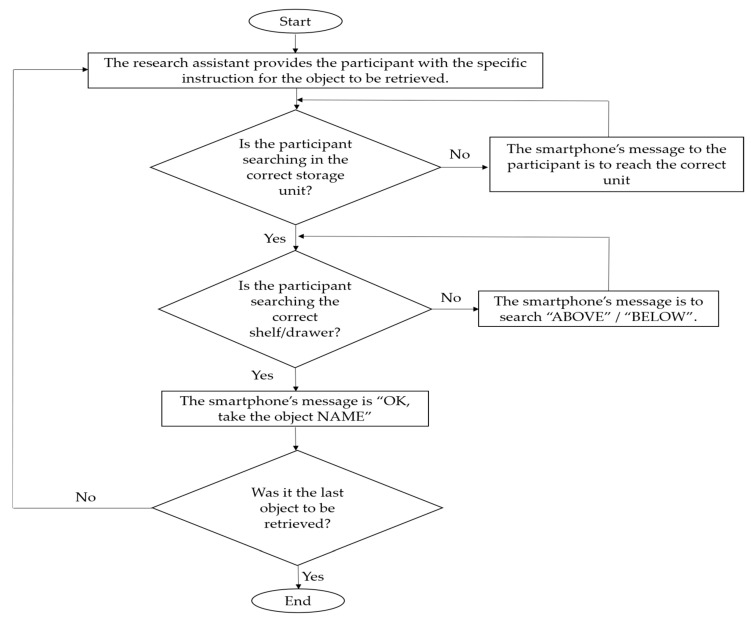
A flowchart illustrating how the technological system worked within a session.

By searching with the hand on the shelves of the cupboard and cabinet or by introducing the hand to the drawers of the drawer unit, the participants’ tag reader (in particular the section with the NFC module) came close to one or more tags of the series available there. The tag reader recognized the tags’ code and sent a signal to the smartphone. The smartphone, which was programmed via the MacroDroid, delivered one of three possible messages. If the participants were searching in the wrong storage unit (this was known to the system because the tag reader had detected tag codes not belonging to the intended unit), the smartphone’s message to the participants was to reach the correct unit. If the participants were in the correct unit but were searching in a wrong shelf or wrong drawer (this was known to the system because the tag reader had detected the tag code of an object type located in that unit, but in a higher or lower shelf/drawer), the smartphone’s message was search “ABOVE” or search “BELOW”. If the participants were in the correct unit and were searching in the correct shelf or drawer (this was known to the system because the tag reader had detected the tag code of the object to be retrieved), the smartphone’s message was “OK, take the object NAME”. Figure 3 illustrates how the technological system worked within a session.

### 2.5. Control Strategy

The control strategy used for Travis and Asher consisted of having small object replicas attached to the outside of each drawer or at the corner of each shelf. The object replicas were to signal the objects available inside the drawers or on the shelves. The control strategy used for Lucas differed from that used for the other two participants only in that the small object replicas were replaced by stickers with the object names written in Braille.

### 2.6. Experimental Conditions and Data Analysis

**Figure 4:**
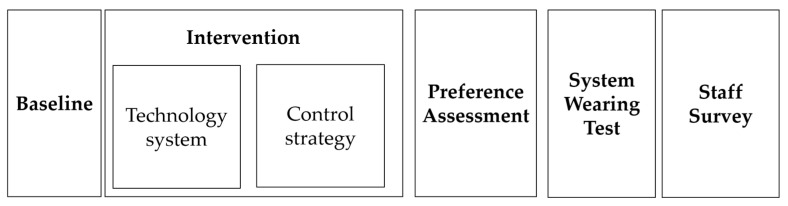
Sequence of the study phases.

The study started with a baseline phase that included different numbers of sessions for the three participants, as in a multiple baseline design across individuals [32]. The baseline phase was followed by an intervention phase in which sessions with the technology system and sessions with the control strategy were regularly alternated for each participant according to an alternating treatments design [32]. This within-subjects design was chosen because it allowed us to compare the effects of the two intervention conditions (i.e., in terms of number of objects retrieved correctly per session and time required for retrieving them) for each participant individually. In addition to this evaluation, the study also included (a) a preference assessment (i.e., the participants were asked to choose whether they wanted to use the system or the control strategy before the start of the sessions), (b) a system-wearing test (i.e., a test to determine whether the participants could independently put on and off the system with its two sections glued together; see Figure 2b), and (c) a staff survey in which staff personnel were asked to rate the technological system. Figure 4 shows the sequence of the study phases.

To ensure that the research assistants’ implementation of the experimental conditions during the sessions was correct (i.e., to ensure high procedural fidelity), feedback was provided to them [33]. Specifically, a research coordinator who had access to video recordings of the sessions informed the research assistants as to whether their performance was accurate.

The “Percentage of data points Exceeding the Median” (PEM) method [34,35] was used to compare the data of the intervention sessions carried out with the technological system and the data of the intervention sessions carried out with the control strategy. The PEM method (a) is a basic and practical tool to evaluate within-subject research data and (b) served to determine for each participant the percentage of intervention sessions with the technological system, in which the number of objects correctly retrieved was higher than the median computed for the sessions with the control strategy. The same method was also used to compare the times required for retrieving the objects during the two types of intervention sessions.

#### 2.6.1. Baseline

During each of the 8–14 baseline sessions, the participants were to retrieve a total of eight objects distributed in the three storage units available in the room and bring those objects to the desk in front of those units. No technological system or control strategy were in use. For each object, the participants received a specific instruction from the research assistants (e.g., “Take a short-sleeved shirt from the cupboard”). The participants were allowed 1.5 min to obtain the object. If they did not manage to do so in the allowed time, the research assistants provided guidance.

#### 2.6.2. Intervention with the System

During the 21–45 intervention sessions with the system, all conditions were as in the baseline except that the participants were provided with the technological system. This was put on (i.e., strapped on the participants’ wrists and backs of their fingers; see Figure 2a) by the research assistants. For each object to be retrieved, the research assistants provided the participants with a specific instruction (see Baseline) and the smartphone with the related object/tag code. The intervention sessions were preceded by three practice sessions in which the participants were helped to familiarize themselves with the use of the system.

#### 2.6.3. Intervention with the Control Strategy

During the 21–45 intervention sessions with the control strategy, the conditions were as in the baseline except that the participants could rely on the presence of mini object replicas (Travis and Asher) or stickers with Braille words (Lucas) on the outside of each drawer or at the corner of each cupboard and cabinet’s shelf. At the start of each session, the participants were reminded to pay attention to those mini object replicas or stickers. The intervention sessions were preceded by three practice sessions in which the participants were helped to use the mini object replicas and stickers.

#### 2.6.4. Preference Assessment

At the start of each of the 17–24 sessions employed for preference assessment, the research assistants asked the participants whether they wanted to use the technological system or the mini objects or stickers of the control strategy. The session was then conducted in line with their choice (i.e., applying the conditions available for the intervention with the system or the intervention with the control strategy).

#### 2.6.5. System Wearing Test

Nine sessions were available for this test. These sessions differed from the aforementioned intervention sessions with the system in that the participants (a) were to use the system with the two sections glued together and the elastic band (see Figure 2b), and (b) were asked to put it on and eventually take if off by themselves. The first of these sessions was preceded by seven to nine practice trials in which the participants were to put the system on and take it off.

#### 2.6.6. Data Recording

Data recording concerned the (a) number of objects correctly retrieved in each session (i.e., in relation to the eight requests made by the research assistants), (b) the time needed to retrieve the eight objects, (c) choices occurred at the start of the preference assessment sessions, and (d) independence or dependence in putting on and taking off the system during the system wearing test. Objects correctly retrieved were those that matched the research assistants’ instructions and were found independent of any research assistants’ guidance. The time to retrieve the objects was computed by summing the intervals between each instruction to retrieve an object and the participants finding/retrieving that object. Interrater agreement was assessed in (a) over 30% of the baseline and intervention sessions, (b) over 50% of the sessions used for assessing preference, and (c) all the sessions used for the system wearing test by having a reliability observer record the data from videos of the sessions. The percentage of agreement on the first two measures (computed by dividing the number of sessions in which the research assistant and the reliability observer reported the same number of objects correctly retrieved and the time for retrieving the eight objects differing less than 25 s by the total number of sessions in which the reliability observer was available) was within the 90–100 range for all participants. The percentage of agreement on recording the last two measures was 100.

#### 2.6.7. Staff Survey

Twenty rehabilitation staff persons (19 women and 1 man with a mean age of 38 years) who worked with people with intellectual and sensory disabilities were involved in the survey. They were first shown a 6 min video which provided clips of the participants retrieving objects with the technology system. Then, they were presented a sheet of paper with three questions concerning the effectiveness of the system, its friendliness to the participants, and its applicability in daily contexts. They were to answer each question by ticking one of the scores available for it. The scores ranged from 1 (most negative) to 5 (most positive).

## 3. Results

**Figure 5:**
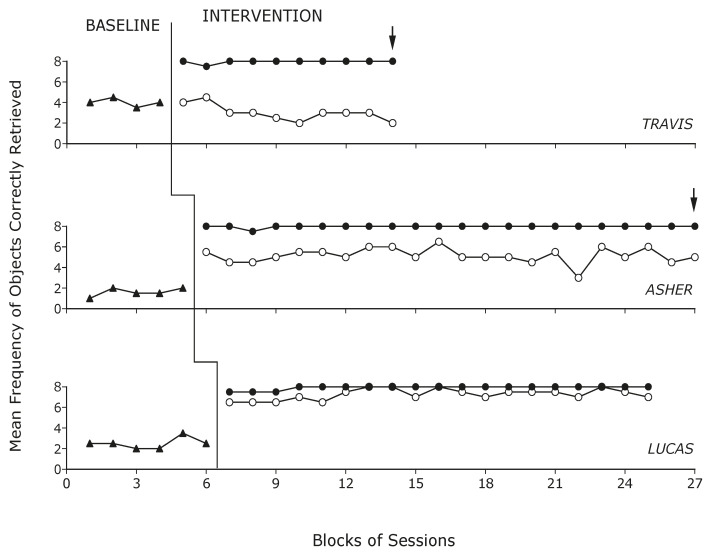
The black triangles indicate the participants’ mean frequency of objects correctly retrieved per session over blocks of baseline sessions. Black and empty circles indicate the participants’ mean frequency of objects correctly retrieved per sessions over blocks of intervention sessions with the technology system and with the control strategy, respectively. The blocks include two sessions (three sessions if an arrow is present).

Figure 5 summarizes the participants’ performance during the baseline and the intervention. The black triangles indicate the participants’ mean frequency of objects correctly retrieved per session over blocks of baseline sessions. Black and empty circles indicate the participants’ mean frequency of objects correctly retrieved per session over blocks of intervention sessions with the technological system and with the control strategy, respectively. The blocks included two sessions (three sessions if an arrow is present).

During the baseline phase, the participants’ mean frequencies of objects correctly retrieved per session (out of the eight that they were instructed to retrieve) were 4, 1.6, and 2.5, respectively. During the intervention sessions with the technological system, the mean frequency per session was nearly eight for all participants. Specifically, Travis made one error over 21 sessions, Asher one error over 45 sessions, and Lucas three errors over 38 sessions. During the intervention sessions with the control strategy, the mean frequencies per session were 3, 5.2, and 7.3 for the three participants, respectively. Comparisons of the intervention frequencies with the technological system and the control strategy, using the PEM method, produced an index of 1 for Travis and Asher and an index of 0.91 for Lucas (indices confirming a positive impact of the technology system). Indeed, all session frequencies with the technological system (Travis and Asher) or all but three of those frequencies (Lucas) were above the median frequency of the intervention with the control strategy.

The mean session times needed to retrieve the eight objects with the technological system were 2.5, 3, and 3.2 min for the three participants, respectively. Their mean session times for retrieving the objects with the control strategy were 2.3, 3.4, and 4.6 min. Comparisons of the session times with the system and the control strategy, using the PEM method, produced indices of 0.62, 0.77, and 0.89 for the three participants, respectively, with the index of the last participant indicating a clear difference in favor of the technological system.

During the preference assessment, Travis chose to carry out all 18 sessions available with the technological system. Asher chose to carry out 15 of the 17 sessions available with the technological system and 2 with the control strategy. Lucas chose to carry out 21 of the 24 sessions available with the system and 3 with the control strategy. In each of the sessions carried out with the technological system, the frequency of objects correctly retrieved was eight (i.e., no errors occurred). The mean frequencies in the sessions carried out with the control strategy were 6 (Asher) and 7.3 (Lucas). The system-wearing test showed that all three participants put on and took off the system with the two sections glued together and the elastic band in each of the sessions independently.

The staff survey provided mean scores of 4.6, 4.1, and 4.3 on the questions concerning the effectiveness of the system, its friendliness to the participants, and its applicability in daily contexts, respectively. Scores of 4 or higher indicate a positive view of the staff persons being surveyed on the questions presented to them.

## 4. Discussion

The results, which are to be taken with caution, given the preliminary scope of this study, suggest that the technological system tended to be more effective than the control strategy in helping the participants to retrieve familiar objects (i.e., in increasing the frequency of objects correctly retrieved or both increasing such frequency and reducing the time needed for retrieving the objects). The results also showed that (a) the participants preferred to use the technological system over the control strategy, (b) were able to put on and take off the system independently, and (b) staff rated the system positively. In light of the results, a few considerations are in order.

First, the system seemed to be quite effective in supporting the participants throughout the study, as shown by the minimal number (or virtual absence) of errors. This suggests that the system was easy to use for the participants (due to the user-friendly interface, and smooth and rapid communication of the system components), did not require the participants to undergo any particular (difficult) learning or adjustment phase, and minimized any possible frustration and anxiety connected to retrieval difficulties or failure.

Second, the fact that the data on the effectiveness of the technology system are in line with the data on the participants’ preferences and staff rating is critically important for the acceptability and applicability of the system [33,36,37,38]. In essence, the participants’ preference for the system could be taken to suggest that they found the system helpful and accepted it as a support tool. The staff’s high rating of the system could be taken to suggest that they considered it a valuable resource that might be adopted in daily contexts.

Third, the possibility of fixing the sections of the system together and the ability of the participants to put the system on and take it off independently constitute relevant evidence from a practical standpoint [39,40]. Indeed, the participants’ ability to access the system without any staff help made the system a tool that the participants of this study and people with similar characteristics could use within their daily context with positive implications for their engagement freedom and quality of life [41,42]. To achieve complete independence, the participants should learn to inform the system as to the object they want to retrieve before starting any search (i.e., learn to do what the research assistants did for them during the study; see Section 2.6.2). A way to achieve this might involve bringing a small object replica fitted with a specific tag in touch with the smartphone (so as to alert it about the object to be retrieved) before starting the search.

Fourth, in light of the above, the system might be seen to represent a resource that the participants can use in the long-term to retrieve objects from a variety of storage units within their context [43,44,45,46]. Such an extension in the use of the system could be realized (a) by fitting the storage units being involved with tag series (i.e., one tag series for each of the different types of objects available) and (b) by programming the smartphone to provide feedback/guidance.

Fifth, the system is not commercially available, but relies on the use of easily accessible components, such as inexpensive tags and a basic smartphone. Obviously, the smartphone needs to be programmed so that it becomes capable of translating the inputs received from the tag reader into specific feedback/guidance for the participants. Programming could be realized via a commercial application, such as MacroDroid v5.43 PRO. With regard to the tag reader, it may be pointed out that one of its components/sections concerns an NFC module that is easy to access commercially. The other section concerns a specific electronic circuit (not commercially available) that uses the input from the NFC module to send signals/messages to the smartphone. The present tag reader was designed to be easily wearable in line with the consensus on the importance of wearable technology [47,48].

### Limitations and Future Research

The three main limitations of this study concern the small number of participants, the lack of maintenance data, and the use of the system over a relatively small set of storage units. The first limitation prevents one from making specific statements about the generality of the results obtained and calls for new studies with additional participants [49,50,51]. The new studies could be conceived as direct or systematic replication studies. In the latter case, variations could be introduced with regard to the technology or the contexts adopted.

The second limitation (lack of maintenance data) prevents one from drawing conclusions as to the long-term effects of the technological system and asks for new studies to extend the data-collection period. Notwithstanding this limitation, one would expect participants to remain motivated to use the system and benefit from it given that it is easily wearable and more effective than (preferred by the participants over) conventional strategies.

The third limitation (use of a small set of storage units) prevents one from making general statements about the possibility of using the system across a variety of storage units and/or across different rooms within the daily context. To address these questions, new studies would need to be arranged with the use of a plurality of storage units distributed in different familiar rooms of the participants’ daily context. Positive data in these studies (together with the results obtained in the preference assessment and the staff survey) would constitute a strong and realistic basis for effective and functional use of the system within daily contexts [52,53].

A fourth possible limitation concerns the fact that the technology involved the use of two applications, the Talking Hands and the MacroDroid applications. To enhance the system’s usability, it could be beneficial to develop a single application capable of both reading tags and providing feedback messages.

## 5. Conclusions

The results, which need to be taken with caution, given the preliminary scope of the study, seem to be encouraging as to the possibility of developing a portable technological system to assist people with intellectual disability and blindness in retrieving objects from different storage units. Based on this early evidence, such a system might be perceived as a potentially relevant assistive device for helping people with special needs in locating, discriminating, and retrieving objects commonly used in daily life. Definite statements about the technological system’s possible impact and overall usability, however, need to be postponed until the limitations of the present study are addressed and new substantial evidence is available. One might also expect new research to upgrade the system and make it more easily available to rehabilitation settings and residential contexts.

## Data Availability

Data are available on request.
